# Genome-wide transcriptome analyses of developing seeds from low and normal phytic acid soybean lines

**DOI:** 10.1186/s12864-015-2283-9

**Published:** 2015-12-18

**Authors:** Neelam R. Redekar, Ruslan M. Biyashev, Roderick V. Jensen, Richard F. Helm, Elizabeth A. Grabau, M. A. Saghai Maroof

**Affiliations:** Department of Crop and Soil Environmental Sciences, Virginia Tech, 185 AgQuad Lane, 24061 Blacksburg, VA USA; Department of Biological Sciences, Virginia Tech, Life Science I building, 24061 Blacksburg, VA USA; Department of Biochemistry, Virginia Tech, Life Science I building, 24061 Blacksburg, VA USA; Department of Plant Pathology, Physiology, and Weed Science, Virginia Tech, Price Hall, 24061 Blacksburg, VA USA

**Keywords:** Phytic acid, *myo*-inositol phosphate synthase, Multidrug-resistance protein ABC transporter, Seed development, Transcriptomics, Differential gene expression, Functional enrichment, Apoptosis, Photosynthesis

## Abstract

**Background:**

Low phytic acid (*lpa*) crops are potentially eco-friendly alternative to conventional normal phytic acid (PA) crops, improving mineral bioavailability in monogastric animals as well as decreasing phosphate pollution. The *lpa* crops developed to date carry mutations that are directly or indirectly associated with PA biosynthesis and accumulation during seed development. These *lpa* crops typically exhibit altered carbohydrate profiles, increased free phosphate, and lower seedling emergence, the latter of which reduces overall crop yield, hence limiting their large-scale cultivation. Improving *lpa* crop yield requires an understanding of the downstream effects of the *lpa* genotype on seed development. Towards that end, we present a comprehensive comparison of gene-expression profiles between *lpa* and normal PA soybean lines (*Glycine max*) at five stages of seed development using RNA-Seq approaches. The *lpa* line used in this study carries single point mutations in a *myo*-inositol phosphate synthase gene along with two multidrug-resistance protein ABC transporter genes.

**Results:**

RNA sequencing data of *lpa* and normal PA soybean lines from five seed-developmental stages (total of 30 libraries) were used for differential expression and functional enrichment analyses. A total of 4235 differentially expressed genes, including 512-transcription factor genes were identified. Eighteen biological processes such as apoptosis, glucan metabolism, cellular transport, photosynthesis and 9 transcription factor families including WRKY, CAMTA3 and SNF2 were enriched during seed development. Genes associated with apoptosis, glucan metabolism, and cellular transport showed enhanced expression in early stages of *lpa* seed development, while those associated with photosynthesis showed decreased expression in late developmental stages. The results suggest that *lpa*-causing mutations play a role in inducing and suppressing plant defense responses during early and late stages of seed development, respectively.

**Conclusions:**

This study provides a global perspective of transcriptomal changes during soybean seed development in an *lpa* mutant. The mutants are characterized by earlier expression of genes associated with cell wall biosynthesis and a decrease in photosynthetic genes in late stages. The biological processes and transcription factors identified in this study are signatures of *lpa*-causing mutations.

**Electronic supplementary material:**

The online version of this article (doi:10.1186/s12864-015-2283-9) contains supplementary material, which is available to authorized users.

## Background

Soybean (*Glycine max* (L.) Merr.) seed is one of the most important agricultural commodities produced worldwide, generating oils, proteins, and carbohydrates [1]. Final seed composition is influenced by both the genotype and environmental factors [2–5]. Breeding programs endeavor to improve the functional properties, and hence the economic value of soybean by reducing anti-nutritive seed components such as phytic acid. Phytic acid (PA), a major source of phosphorus in seeds, can cause problems such as poor mineral bioavailability and phosphate pollution [6, 7]. Low PA (*lpa*) crops are therefore highly desirable for reducing anti-nutritional and environmental effects of conventional crops [8–10]. The *lpa* soybean line ‘V99-5089’ carries a non-lethal, recessive mutation in the *myo*-inositol phosphate synthase (MIPS) 1 gene, whereas ‘CX-1834’ carries mutations in two multidrug resistant protein (MRP) genes, encoding ATP-binding cassette transporters [11–16]. The MIPS1 gene, expressed during seed development in soybean, is associated with the conversion of glucose-6-phosphate to *myo*-inositol-3-*mono*phosphate, which is the first step in PA biosynthesis pathway [17, 18]. Loss of function mutation in this gene disrupts this pathway. The MRP genes are also highly expressed in developing embryos, however, the mechanism by which they regulate PA levels in soybean is poorly understood [13].

The PA biosynthesis pathway plays a vital role in maintaining homeostasis. Several pathway intermediates, such as *myo*-inositol-1,4,5-trisphosphate, act as secondary messengers in signal transduction and are known to regulate growth and developmental processes, including phosphorus and mineral storage, DNA repair, chromatin remodeling, RNA editing and export, ATP generation, regulation of gene expression, regulation of guard cells, auxin metabolism and cell-wall polysaccharide biosynthesis [17, 19–24]. Numerous studies have reported the effects of *lpa* on plant growth and development. An RNAi-mediated *mips1* knockdown in soybean was reported to inhibit seed development along with reduced PA content [25]. Similarly, seed embryo defects were reported for Arabidopsis and common bean (*Phaseolus vulgaris L.*) *mips* mutants [26, 27]. The *mips* mutation regulates raffinose family oligosaccharide pathway, and mutants exhibit impaired pathogen resistance, programmed cell death in leaves, and polar auxin transport causing deformed cotyledon development [26, 28–33]. The *mrp* mutants are known to exhibit *lpa* phenotypes in soybean, rice, maize, and Arabidopsis [12, 13, 34, 35]. MRP knockout studies in Arabidopsis exhibit phenotypes such as insensitivity to abscisic acid-mediated germination and unresponsive stomata opening, resulting in reduced transpiration rate and increased drought tolerance; which were rescued by MRP overexpression [36]. Finally, *lpa* crops are known to show poor seedling emergence, resulting in reduced crop yield, which decreases the agronomic value of *lpa* crops [37–39].

Despite these diverse physiological responses of different *lpa* mutations, very little is known about the effect of combining *lpa* mutations together on seed development and the underlying regulation of gene expression in soybean. Bowen et al. [40] investigated microarray-based gene expression changes in developing embryos of barley *lpa* mutant. This study identified several differentially expressed genes associated with different cellular processes, such as carbohydrate metabolism, hormonal signaling and transport [40]. Recent developments in sequencing technologies have enhanced the scope of genome-wide gene expression studies to a level far beyond microarray. An advanced generation recombinant inbred line (RIL) *3mlpa* with three *lpa*-causing mutations, derived from a bi-parental cross of V99-5089 and CX-1834, provides a higher reduction in PA content of soybean seed relative to those with single mutations [41]. The *3mlpa* triple mutant line and another advanced generation RIL 3MWT without any *lpa*-causing mutations, derived from the same cross (Table 1) are unique genetic materials for gene expression studies. In this report, we used mRNA-sequencing (RNA-Seq) to study the effect of *lpa*-causing mutations from MIPS1 and MRP genes on global changes in gene expression profiles of developing soybean seeds. A total of 30 transcriptome datasets derived from five developing seed stages with three biological replicates each of *3mlpa* and 3MWT soybean line were sequenced and analyzed. To the best of our knowledge, this is the first extensive report describing the gene regulatory effect of MIPS1 and MRP mutations together. The significantly enriched biological processes and transcription factors identified in this study further our understanding of seed development *lpa* mutants.

**Table 1:** Characteristics of experimental lines and their parents

Soy Lines	Genotype	Phytate	Emergence	Stachyose	Sucrose
V99-5089	*mips1/*MRP-L/MRP-N	Low	Low	Low	High
CX-1834	MIPS1*/mrp-l/mrp-n*	Low	Low	Normal	Normal
*3mlpa*	*mips1/mrp-l/mrp-n*	Low	Low	Normal	Normal
3MWT	MIPS1/MRP-L/MRP-N	Normal	Normal	Normal	Normal

All experimental lines represented here are homozygous. Italicized text indicates mutations or mutant line. The soybean lines *3mlpa* and 3MWT are two recombinant inbred lines derived from an advanced generation of a cross of V99-5089 with CX-1834

## Results and discussion

### Differential expression analyses

**Fig. 1:**
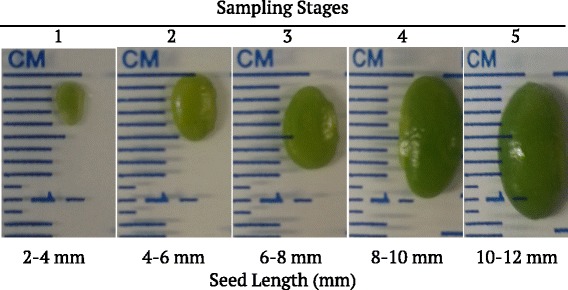
Seed developmental stages for sampling. Three biological replicates sampled per stage for both *3mlpa* and 3MWT

**Fig. 2:**
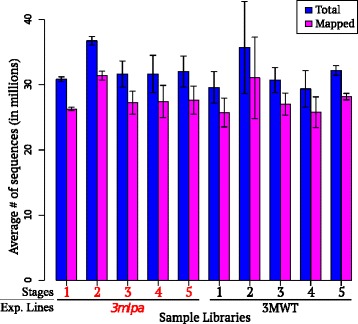
Alignment Statistics. Average number of sequences generated and mapped to reference genome for each library. Total of 30 libraries were sequenced for this experiment. Error bars indicate standard error for biological replicates

**Fig. 3:**
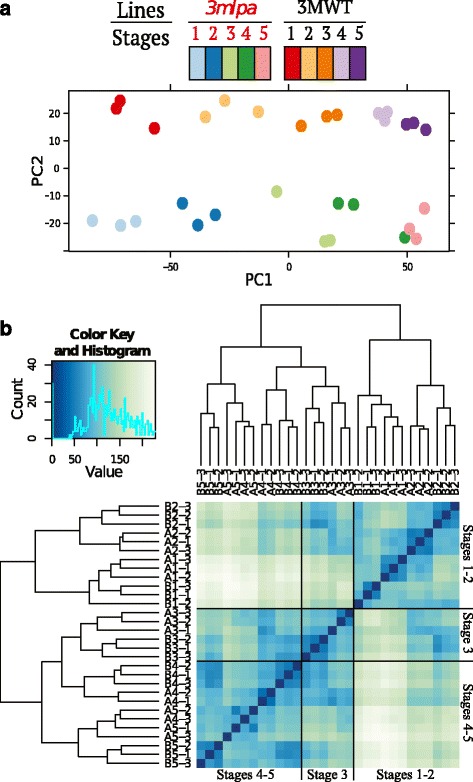
Biological sample variability. **a** Principle component analysis plot explains the variance in gene expression data from biological sample libraries along PC1 or X-axis and PC2 or Y-axis. **b** Sample clustering heatmap representing sample-to-sample distance. Blue color suggests similarity between sample libraries. Samples A and B correspond to *3mlpa* mutant, and 3MWT, respectively, e.g. A3-2, means *3mlpa*-stage3-replicate2 (See Additional file 3 for more information)

Whole soybean seeds comprised of cotyledons, endosperm, and seed coat, were sampled in triplicate from *3mlpa* and 3MWT lines at five stages of seed development (Fig. 1). These stages were chosen to correspond to seed filling stages post embryo development and before desiccation [2]. More than 961 million sequencing reads were obtained from 30 mRNA libraries, and 86.69 % (more than 833 million) of the reads were mapped to the annotated soybean reference, Williams 82 genome assembly 1.0 sequence (Fig. 2, Additional file 1). Read counts (number of reads mapping to a given gene) were estimated from the sequence mapping data for all the annotated gene models (total of 54,175) of Williams 82 genome (annotation v1.1). Normalized read count data were used for differential expression analyses. Principal component analysis (PCA) and sample-to-sample distance clustering variance stabilized *log*_*2*_ transformed normalized read count data for all genes from 30 sample libraries, are shown in Fig. 3. Sample libraries generated from different seed developmental stages were distinctly represented along PC1, in a unidirectional pattern starting from stage 1 to 5 in PCA; whereas those generated from the *3mlpa* line were clearly differentiated from their respective 3MWT libraries along PC2 (Fig. 3a). This means that first and second major contributors to variation in the data are seed developmental stages and genotypes, respectively. At the same time, the biological replicates of each stage clustered together, suggesting minimal variance between replicates. Similarities and dissimilarities between individual sample libraries were visualized using heatmap of sample-to-sample distance clustering as shown in Fig. 3b. The sample libraries from early (stages 1–2) and late (stages 4–5) seed development stages were dissimilar to each other, while those from stage 3 are partially similar to both groups.

**Fig. 4:**
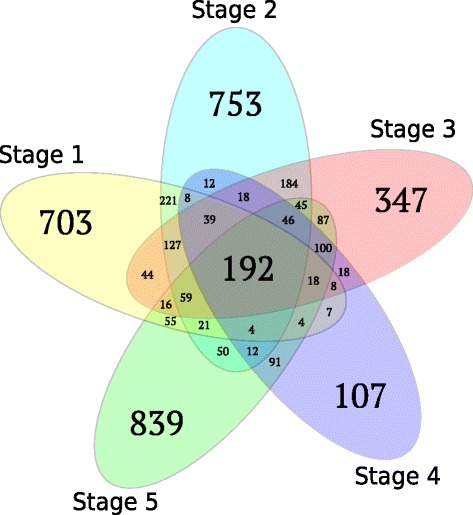
Overlap of differentially expressed genes between developmental stages. Each oval in this Venn diagram corresponds to a seed developmental stage. Numbers within each oval represents significant differentially expressed genes between *3mlpa* and 3MWT overlapping between- or unique to-seed developmental stages

We identified a total of 6988 (4235 unique) genes with significant differential expression between the *3mlpa* and 3MWT lines at five seed developmental stages (Table 2). Some of these DEGs may be due to non-isogenic background of the experimental lines. Of these differentially expressed genes (DEGs), 3321 (47.5 %) and 3493 (48.5 %) genes were identified as up- and down-regulated in the *3mlpa* line. About 174 (2.5 %) and 102 (1.5 %) genes were expressed only in either *3mlpa* line or 3MWT, respectively (Table 2). Several genes were differentially expressed in more than one stage, and 192 genes were differentially expressed between *3mlpa* versus 3MWT in all five stages of seed development (Fig. 4).

**Table 2:** Differential gene expression between *3mlpa* and 3MWT

Stages	1	2	3	4	5
Differentially expressed genes (DEGs)	1526	1791	1348	684	1639
DEGs up-regulated in *3mlpa*	831	1114	788	269	493
DEGs down-regulated in *3mlpa*	695	677	560	415	1146
DEGs only expressed in *3mlpa*	29	41	39	32	33
DEGs only expressed in 3MWT	26	20	17	21	18

Out of total 6988 DEGs identified in five seed developmental stages, 4235 were unique, meaning only counted once. Remaining genes were repeatedly identified in more than one stage

### Functional enrichment analyses

Functional enrichment of gene ontology (GO) and transcription factor (TF) families employing DEGs for each stage was performed using a statistical hyper-geometric test employing the Benjamini-Hochberg method for multiple testing to obtain *adjusted*-P-values. The enriched results were then filtered using *adjusted-*P-value < =0.01 (or 1 % FDR) to obtain highly significant enriched terms. Table 3 shows highly significant enriched GO terms identified at five seed developmental stages. Although some terms were found in more than one stage or were stage-specific, none were common in all five stages. In order to simplify the interpretations, we grouped the stages into early (stages 1–2) and late phase (stages 4–5) of seed development. Enriched GO terms from stage 3, showed partial overlap with both early and late phases. Most of the DEGs associated with significantly enriched biological processes in early phase of seed development were up-regulated, whereas those in late phase were down-regulated in *3mlpa* line.

**Table 3:** Enriched gene ontology terms associated with biological processes

Stage	GO term	Biological processes	DEG	*P*-value	FDR
1	GO:0006915	Apoptosis	31	4.0E-04	3.4E-03
	GO:0006073	Cellular Glucan Metabolic Process	17	6.8E-05	7.4E-04
	GO:0006334	Nucleosome Assembly	15	6.9E-06	1.2E-04
	GO:0006412	Translation	71	2.9E-10	6.4E-08
2	GO:0006915	Apoptosis	39	1.1E-05	7.9E-04
	GO:0006073	Cellular Glucan Metabolic Process	18	1.0E-04	3.4E-03
	GO:0006857	Oligopeptide Transport	15	1.6E-04	3.5E-03
	GO:0055114	Oxidation Reduction	140	3.6E-06	7.9E-04
	GO:0055085	Transmembrane Transport	68	3.4E-04	6.3E-03
3	GO:0006915	Apoptosis	42	1.9E-10	1.7E-08
	GO:0006096	Glycolysis	10	1.2E-03	9.8E-03
	GO:0045087	Innate Immune Response	20	2.2E-06	5.1E-05
	GO:0015979	Photosynthesis	20	6.0E-07	2.2E-05
4	GO:0006091	Generation of precursor metabolites and energy (Glycolysis and Photosynthesis)	14	7.0E-06	4.9E-04
	GO:0015979	Photosynthesis	16	4.1E-09	5.6E-07
5	GO:0008652	Cellular Amino acid Biosynthetic Process	18	1.0E-03	4.0E-03
	GO:0006096	Glycolysis	12	7.3E-04	3.8E-03
	GO:0006184	GTP Catabolic Process	7	6.6E-04	3.6E-03
	GO:0008610	Lipid Biosynthetic Process	30	9.2E-04	3.8E-03
	GO:0006108	Malate Metabolic Process	6	8.3E-05	5.7E-04
	GO:0006334	Nucleosome Assembly	20	9.2E-09	2.1E-07
	GO:0015979	Photosynthesis	55	4.4E-30	1.1E-27
	GO:0009765	Photosynthesis, Light Harvesting	14	1.3E-09	6.6E-08
	GO:0006814	Sodium ion Transport	5	8.2E-04	3.8E-03
	GO:0006414	Translational Elongation	9	4.6E-04	2.7E-03
	GO:0006412	Translation	63	1.7E-06	1.9E-05

The GO terms identified in this study are more specialized (or, child) terms in the GO hierarchy

**Fig. 5:**
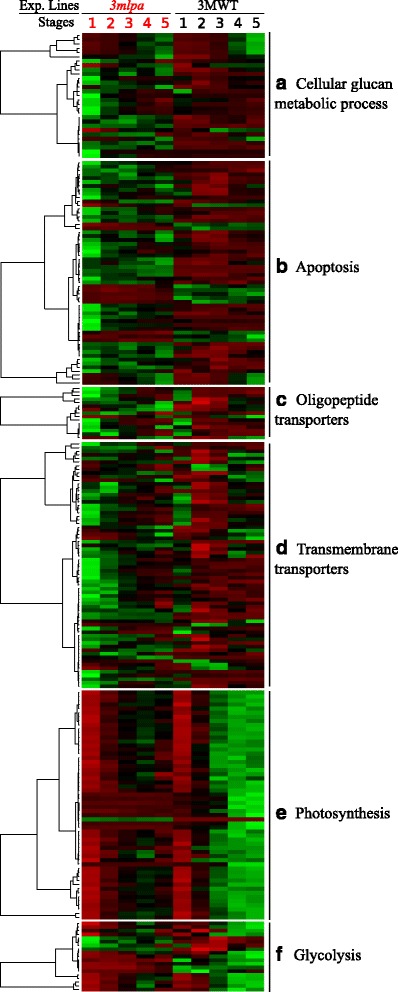
Mean normalized gene expression profiles of DEGs associated with biological processes (**a**) Cellular glucan metabolic process, (**b**) Apoptosis, (**c**) Oligopeptide transporters, (**d**) Transmembrane transporters, (**e**) Photosynthesis, and (**f**) Glycolysis. Hierarchical clustering of mean normalized gene expression values based on euclidean distance between seed developmental stages of *3mlpa* and 3MWT. Rows represent genes, while columns represent samples. Green color indicates higher gene expression values

**Fig. 6:**
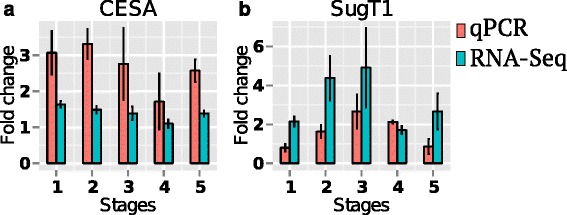
Relative gene expression of DEGs for RNA-Seq data validation. Fold change between *3mlpa* and 3MWT at respective seed developmental stages for genes encoding (**a**) Cellulose synthase (CESA: Glyma12g36570) and (**b**) Sugar transporter (SugT1: Glyma08g06420). Green and orange bars indicate mean fold change values from RNA-Seq and qPCR experiments, respectively. There was no significant difference in the gene expression profiles estimated using qPCR and RNA-Seq analyses at significance level of 0.01 (See Additional file 8 for more information)

Hierarchical clustering of mean normalized gene expression levels of DEGs associated with the enriched biological processes of cellular glucan metabolism, apoptosis, cellular transport, photosynthesis and glycolysis is shown in Fig. 5. The biological processes of cellular glucan metabolism, apoptosis and cellular transport were up-regulated in early stages (Fig. 5a-d), while those associated with photosynthesis and glycolysis were down-regulated in late stages (Fig. 5e-f) of *3mlpa* line. The relative gene expression data from RNA-Seq analysis for two randomly selected DEGs (CESA: Glyma12g36570, and SugT1: Glyma08g06420) were evaluated using qPCR (Fig. 6). The genes CESA and SugT1 are associated with cellular glucan metabolism and cellular transport respectively. The RNA-Seq analysis showed up-regulation of CESA and SugT1 genes in *3mlpa* in early stages of seed development. Both real time quantification and RNA-Seq analysis showed similar expression pattern for the CESA and SugT1 genes. There was no significant difference in the gene expression data obtained from qPCR and RNA-Seq analyses at the 0.01 significance level. We also tested relative expression of MIPS1, MRP-L and MRP-N genes, which were not differentially expressed in any of our seed stages. Both qPCR and RNA-Seq analyses estimated similar fold change expression with no significant difference between them (Additional file 2: Figure S1).

Soybean genome assembly 1 is annotated to contain 5683 TF genes from 63 TF families [42]. Total of 512 differentially expressed TF genes were identified in this study. These genes belonged to 32, 31, 33, 20, and 34 different TF families in seed developmental stages 1 to 5 respectively. TF family enrichment analysis resulted in identification of 2, 2, 4, 2, and 2 TF families significantly enriched in seed developmental stages 1 to 5, respectively (Table 4). We observed TF families GRAS (**G**ibberellin-Insensitive, **R**epressor of ga1–3, **S**carecrow), WRKY, ZF-HD (**Z**inc **F**inger-**H**omeodomain), and ZIM (**Z**inc-finger protein expressed in **I**nflorescence **M**eristem) were enriched in early stages (stages 1–2), whereas, families CAMTA (**CAlM**odulin-binding **T**ranscription **A**ctivator), GRF (**G**rowth-**R**egulating **F**actor1), MBF1 (**M**ultiprotein **B**ridging **F**actor 1), SNF2, and TCP (**T**eosinte branched 1, **C**ycloidea, **P**CF) were enriched in later stages (stages 3–5) of seed development. Among these enriched TF families, TCP family was represented in stages 3 and 4, while CAMTA was represented in stages 3–5. All together, these TF families represented 53 unique DEGs. The genes belonging to enriched TF families, such as WRKY, GRAS, ZIM, CAMTA, GRF, and SNF2, were up-regulated, whereas, those belonging to ZF-HD, MBF1B, and TCP were down-regulated in the *3mlpa* mutant.

**Table 4:** Transcription factor families significantly enriched in developing seed stages

Stages	TF family	FDR	Gene symbols	DEGs associated with the TF family	Functions
1	WRKY	4.92E-04	WRKY33, WRKY40, WRKY29, WRKY6, WRKY28, WRKY23, WRKY15, WRKY11	Glyma11g29720, Glyma08g23380, Glyma08g02160, Glyma13g44730, Glyma09g00820, Glyma12g10350 Glyma08g08720, Glyma13g38630, Glyma15g11680, Glyma03g37940, Glyma17g18480, Glyma05g20710, Glyma06g08120, Glyma04g08060	Associated with plant defense, senescence, and abiotic stress [73]
	GRAS	3.19E-04	SCL1, SCL3, SCL5, SCL14, PAT1, SGR7	Glyma04g28490, Glyma18g09030, Glyma08g43780, Glyma11g14670, Glyma14g27290, Glyma13g09220, Glyma15g04160, Glyma14g01960, Glyma14g01020, Glyma17g17400, Glyma13g02840	Involved in gibberellin signaling, phytochrome A signal transduction, controls radial patterning [74–77]
2	ZIM	8.60E-03	JAZ6, JAZ12	Glyma17g04850, Glyma16g01220, Glyma07g04630	Repressor of jasmonate responses [78]
	ZF-HD	7.44E-03	HB22, HB24, HB33	Glyma06g09970, Glyma04g09910, Glyma08g06120, Glyma11g07360, Glyma14g35770	Regulator of ABA-response [79]
3	TCP	3.23E-05	TCP3, TCP20	Glyma09g42140, Glyma16g05840, Glyma19g26560, Glyma12g35720, Glyma06g34330, Glyma13g34690, Glyma03g02090, Glyma09g42120	Controls cell expansion and morphogenesis, negatively regulates auxin response, and promotes flavonoid synthesis [80, 81]
	SNF2	1.63E-03	EDA16, RGD3, PIE1, SYD, CHR5	Glyma07g31180, Glyma09g36910, Glyma02g29380, Glyma17g02540, Glyma13g25310, Glyma02g45000	Embryo sac development, repressor of flowering, chromatin remodeling, gravitrophism [82]
	GRF	8.68E-03		Glyma19g28010	Cotyledon growth [83]
	CAMTA	4.18E-04	CAMTA3, SR1	Glyma05g31190, Glyma08g14370, Glyma17g04310	Negative regulator of plant immunity [51, 84]
4	TCP	9.25E-03	TCP3	Glyma13g34690, Glyma09g42120, Glyma09g42140	Negatively regulates auxin response, and promotes flavonoid synthesis [80]
	CAMTA	8.14E-04	CAMTA3, SR1	Glyma05g31190, Glyma08g14370	Negative regulator of plant immunity [51, 84]
5	MBF1	5.27E-03	MBF1B	Glyma06g42890	Negative regulator of ABA-dependent inhibition of germination [85]
	CAMTA	9.57E-03	CAMTA3, SR1	Glyma08g11080, Glyma08g14370	Negative regulator of plant immunity [51, 84]

### Regulation of defense response in early seed development of *3mlpa* mutant

‘Apoptosis’ (GO:0006915) and ‘Innate immune response’ (GO: 0045087) were enriched in early phases of seed development, particularly in stages 1–3 and stage 3 respectively, with different sets of DEGs identified for these stages (Table 3). While several of these genes are shared between these two categories, a total of 61 DEGs were associated with both. Forty-seven of these genes (77 %) were up-regulated and 14 (23 %) genes were down-regulated in the *3mlpa* mutant line. The FC and *log*_*2*_FC ratio of apoptosis-related differentially expressed genes are reported in Additional file 3. These defense-related DEGs encoded (a) LRR (leucine rich repeat) domain-containing disease resistance proteins, (b) NB-ARC (nucleotide-binding adaptor shared by APAF-1, R proteins, and CED-4) domain-containing disease resistance proteins, (c) Bcl-2-associated athanogene 1, (d) ADR1-L1 (Activated Disease Resistant 1-like 1), (e) cysteine proteinases, (f) protein kinase and (g) NTP hydrolases. Apoptosis is a common phenotype observed in *mips1* mutants of *Arabidopsis thaliana* [26, 30, 43].

The defense-related genes identified in this study lie upstream of the apoptotic processes. For example, the LRR and NB-ARC domain-containing disease resistance genes are involved in initiating the defense response via induction of salicylic acid (SA) and formation of reactive oxygen species (ROS), both of which ultimately leading to cell death. *Arabidopsis thaliana mips1* mutants were shown to accumulate SA and ceramide, both associated with formation of a cell death lesion area, with the phenotype rescued by treating with either *myo*-inositol or galactinol [26, 30, 43]. Apoptosis in *mips1* mutants is also regulated by light intensity and ROS such as peroxisomal hydrogen peroxide and the superoxide anion [26, 30, 43]. *Myo*-inositol mitigates SA-dependent apoptosis and defense responses [43]. Although apoptosis is associated with *mips1* mutation and not with *mrp* mutation, induction of the defense-related genes in *3mlpa* may not be solely due to *mips1* mutation. The ADR1-L1 genes (Glyma14g08700 and Glyma17g36420), which belong to a subgroup of CNL-A clade of coiled-coil NBS-LRR gene family, showed two-fold higher expression in *3mlpa* mutant [44]. The ADR1 genes are positive regulators of SA-mediated cell death and defense response [45]. Enhanced expression of ADR1 gene was previously shown to establish drought tolerance in presence of SA [46]. And interestingly, the *mrp5* mutants of *Arabidopsis* are also known to be drought tolerant [36]. Role of MRP genes in regulating defense responses is still unknown, and further studies are required to confirm the association of the drought tolerant phenotype of the *mrp* mutants and up regulation of ADR1 genes as in *3mlpa*. In addition, several WRKY transcription factor family genes that regulate expression of other defense-related genes were also up-regulated in early stages of seed development in *3mlpa* mutant. In summary, the *lpa*-causing mutations in *3mlpa* mutant may play a role in initiating defense responses during seed development.

### Regulation of photosynthesis in late stages of seed development in *3mlpa* mutant

‘Photosynthesis’ (GO:0015979) was enriched in late phase (stages 3–5) of seed development, with most of the photosynthesis-related DEGs from these stages were down-regulated in *3mlpa* mutant (Table 3, Fig. 5). Fifty-six DEGs were associated with photosynthesis and of these only one gene (Glyma15g40131) was up-regulated in *3mlpa* mutant. These DEGs genes encode different subunits in photosystem (PS) I (psaD, psaE, psaF, psaG, psaH, psaK, psaL, and psaN), and PS II (psbA, psbE, psbP, psbQ, psbW, psbX, and psbY), light-harvesting chlorophyll complex proteins from PS I (LHCA1 and LHCA2) and PS II (LHCB1, LHCB4, LHCB5), and a single gene encoding for magnesium protoporphyrin IX methyl transferase (CHLM) (Additional file 4). A previous study on transcriptome profiles of soybean cultivar ‘Williams’ reported expression of photosynthesis-related genes early in development when seed size was 25–50 mg [47]. This would correspond to stages 2–3 in our investigation. Bowen et al. [40] previously reported two photosynthesis related probesets that were differentially expressed in M955 maize line with *lpa* at 7 days post anthesis. One representing chloroplast nucleoid DNA binding protein was down-regulated, while another representing early light inducing protein, was up-regulated in M955 line [40]. Although these proteins were not identified in our study, regulation of photosynthesis-related genes in *lpa* mutants is a common observation.

The PSI and PSII protein complexes are involved in capturing light energy during photosynthesis. Differential expression of these genes may translate into different numbers of PSI and PSII protein complexes between *3mlpa* and 3MWT. The methyl transferase CHLM is involved in chlorophyll metabolism where it catalyzes the formation of Mg-protoporphyrin IX monomethyl ester [48]. Bruggeman et al. [49] have reviewed the association of chloroplast activity with the formation of cell death lesions, where excess light energy can trigger cell death. Down regulation of photosynthesis-related genes in *3mlpa* may prevent apoptosis by limiting the light energy during late stages of seed development. Cell death lesion formation in *mips1 Arabidopsis* mutants is dependent on light intensity and ROS accumulation [26, 30, 43], and abolishing PSII assembly was shown to reduce cell death lesions [50]. Tetrapyrrole biosynthesis is also regulated to prevent accumulation of ROS, which can also lead to cell death [49]. Moreover, we also identified enhanced expression of CAMTA3/SIGNAL RESPONSIVE 1 (SR1) TF genes in *3mlpa* during late stages of seed development. CAMTA3/SR1 is a negative regulator of SA-mediated plant immunity and it inhibits cell death [51]. Therefore, these observations strongly suggest that the up-regulation of cell death inducing genes in early stages of seed development in *3mlpa* mutant is suppressed in late stages. *Myo*-inositol promotes photosynthesis in *Mesembryanthemum crystallinum* so another explanation for down-regulation of photosynthesis-related genes in *3mlpa* mutant may be the *mips1* mutation, which reduces *myo*-inositol levels [52].

### Regulation of glucan metabolism in early seed development of *3mlpa* mutant

Cellular glucan metabolic process (GO:0006073) was found enriched in both stage 1 and 2 (early phase of seed development), however the DEGs associated with this process were stage-dependent (Table 3, the FC and *log*_*2*_ FC ratios for these genes are reported in Additional file 5). Twenty-seven genes were up-regulated, and two genes (Glyma02g47076 and Glyma04g43470) were down-regulated in *3mlpa* mutant. Six genes were differentially expressed in both stages and all were up-regulated in *3mlpa* mutant. The FC of up-regulated genes ranged between 1.6–60 and 1.3–18 for stages 1 and 2, respectively. In case of down-regulated genes, the FC was between 0.2–0.6 for stages 1 and 2, respectively. Two genes (Glyma09g07070 and Glyma15g18360) showed no expression or zero read count in 3MWT resulting in infinite (∞) value for FC and *log*_*2*_ FC ratio. The differentially expressed genes associated with this process encoded two enzymes: (a) cellulose synthase (also, known as glucan synthase, or CESA, EC 2.4.1.12); and (b) xyloglucan endotransglucosylase/hydrolase (also known as xyloglucan:xyloglucosyl transferase or XET, EC 2.4.1.207). Functional enrichment also showed over-representation of genes associated with XET enzymatic activity (GO:0016762). The CESA and XET enzymes are involved in synthesis of building units of cell wall, i.e. cellulose, and xyloglucan (hemicellulose) chains, respectively. Cellulose synthase activity is an integral component of cell wall synthesis. A previous study on transcriptome profiles of soybean line ‘A81-356022’ reported higher cellulose synthase activity in developing seeds at 25–28 days post flowering [53]. Differential expression of the CESA and XET genes in early seed development suggests regulation of cell wall synthesis in *3mlpa* mutant line. Role of *myo-*inositol in cell wall synthesis via the *myo-*inositol oxidation pathway, resulting in formation precursors of pectin and hemicellulose, is well established and regulation of cell wall biosynthesis in *mips* mutants is therefore expected [23, 54]. The contribution of either the *mips* or *mrp* mutations towards this response lies out of scope of this experiment, however, it is possible that *3mlpa* mutants initiate formation of cell wall polysaccharides at earlier stages of development relative to the non-mutants.

### Regulation of cellular transport in early seed development of *3mlpa* mutant

Processes involving oligo-peptide transporters (GO:0006857) and transmembrane (GO:0055085) transporters were enriched in stage 2 (Table 3). Although several transporter genes were differentially expressed in other stages, the GO term associated with the transporter activity was not significantly enriched in those stages. Together, oligo-peptide and transmembrane transport activity were associated with a total of 79 unique differentially expressed genes and 86 % of these genes were found up-regulated in the mutant line. Twenty-nine (44 %) of these up-regulated genes encode for multidrug transporters, including 15 from major facilitator superfamily (MFS), six from multidrug and toxic compound extrusion (MATE) efflux carrier superfamily, five from multidrug resistance superfamily, two genes for P-glycoprotein (PGP) and 1 gene for ATP binding cassette (ABC) subfamily (B4) (Additional file 6). Most of the multidrug transporters are annotated as being involved in removal of toxic compounds from the cell [55]. Recently, a MFS transporter, Zinc-Induced Facilitator-Like 1 from Arabidopsis was reported to be associated with polar auxin transport in roots, as well as regulation of stomata for drought stress tolerance [56]. The ABC B4 transporters are also involved in auxin-gradient dependent polar auxin transport in root [57], and PGP transporters are involved in cellular and long distance transport of auxin [58]. Defects in *mips* mutant embryos were previously associated with impaired endomembrane system and lack of polar auxin transport [32]. The genes encoding monosaccharide transporters such as Sugar Transport Protein, Inositol Transporter and Polyol/Monosaccharide transporter were also differentially expressed in our dataset. These genes are involved in transport of sugars such as glucose, fructose, galactose, mannose, xylose, sorbitol, mannitol, xylitol, and epimers and derivatives of myo-inositol [59]. Other genes encoding transporters/carriers of cationic amino acids, oligopeptides, potassium, sulfate, nitrates, zinc, chloride and dicarboxylate ions were also identified in *3mlpa* mutant line. Although substrates of MRP-L and MRP-N transporters are still unknown, enrichment of the transporter genes may be compensating for the loss of MRP-L and MRP-N.

### Regulation of raffinose family oligosaccharide biosynthesis in developing seeds of *3mlpa* mutant

**Fig. 7:**
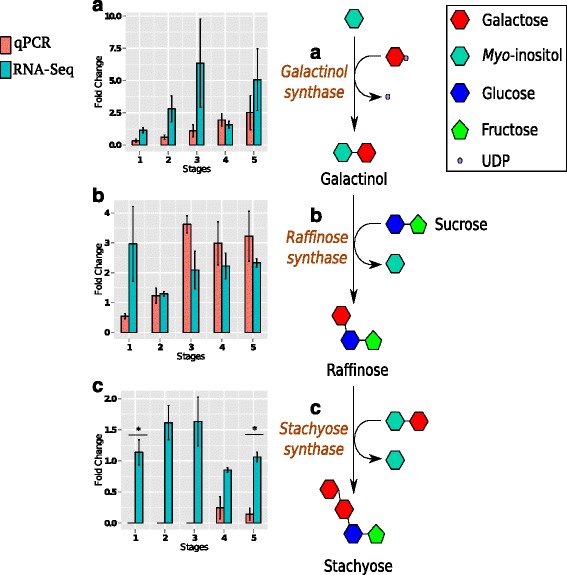
Raffinose family oligosaccharide biosynthesis pathway. Bar graphs indicate fold change (*3mlpa* over 3MWT) ratio of normalized expression values for genes (**a**) galactinol synthase, (**b**) raffinose synthase and (**c**) stachyose synthase at 5 stages of seed development. Green and orange bars indicate mean fold change values from RNA-Seq and qPCR experiments, respectively. Gene expression profiles showing significant difference between qPCR and RNA-Seq analyses are indicated by asterisk (*) (Refer Additional file 8 for more information)

The raffinose family oligosaccharides such as raffinose and stachyose are considered to be anti-nutritive components in seeds [60]. The biosynthesis of raffinose family oligosaccharide (RFO) involves three steps: (a) galactinol synthase (GS) catalyzes the formation of galactinol from *myo*-inositol and UDP-galactose, (b) raffinose synthase (RS) catalyzes the formation of raffinose by adding sucrose to galactinol and releasing a *myo*-inositol molecule, and (c) stachyose synthase (SS) catalyzes the formation of stachyose by adding galactinol to raffinose and releasing *myo*-inositol (Fig. 7). The *mips1* mutation is associated with low stachyose phenotype in the parental line V99-5089 [15]. However, the *3mlpa* mutant shows normal stachyose levels in mature seeds despite having *mips1* mutation [41]. The genes encoding RFO biosynthesis pathway enzymes are expressed in seed filling and dessication stages of soybean seed development [61]. None of these genes showed significant differential expression at 1 % FDR, which may be one possible explanation for normal stachyose phenotype in *3mlpa* mutant. Figure 7 represents RFOs biosynthesis pathway, and the relative gene expression values for selected genes in the pathway.

There are six genes (Glyma03g38080, Glyma03g38910, Glyma10g28610, Glyma19g40680, Glyma19g41550, and Glyma20g22700) annotated as GS in first assembly of reference soybean genome (Glyma.Wm82.a1.0). The Glyma19g41550 gene showed highest-FC in RNA-Seq experiment, so we estimated relative expression for this GS gene using qPCR. The relative transcript levels of GS gene showed a gradual increase in *3mlpa* during seed development (Fig. 7a). RNA-Seq data showed similar gradual increase in expression in *3mlpa* during seed development, but expression dropped at stage 4 (Fig. 7a). Glyma06g18890 encodes RS (also known as RS2 or Rsm1) is expressed during seed development in Williams 82 soybean line, and is known to control the raffinose and stachyose content in soybean seeds [60, 62]. The relative transcript levels of the RS gene showed more than 2-fold change in *3mlpa* in late phase (stages 3–5) of seed development, with no difference between the stages 3–5 (Fig. 7b). The fold change of RS remained below 2 in *3mlpa* during early stages. This validates the RNA-Seq data except for stage 1, where fold change of RS was close to 3 in *3mlpa* (Fig. 7b). Glyma19g40550 is the only SS encoding gene in Glyma.Wm82.a1.0. At stage 1 and 5, SS gene showed significant difference in the fold change obtained from qPCR and RNA-Seq analyses. The relative transcript levels of the SS gene showed almost no change between *3mlpa* and 3MWT with fold change below 2 (Fig. 7c). It is possible that mechanisms other than differential expression are involved in regulation of RFO biosynthesis pathway, which does not allow us to elaborate further on these observations.

## Conclusions

The PA biosynthesis pathway intermediates are involved in many growth and developmental processes. Several genes encoding key enzymes regulating this pathway are mutated to obtain *lpa* crops. We used a transcriptomics approach to compare the gene expression profiles of a *lpa* soybean line (*3mlpa*) carrying three (*mips1, mrp-l,* and *mrp-n*) mutations and a non-mutant line, 3MWT, at 5 stages of soybean seed development. The differential expression and functional enrichment analyses indicated regulation of biological processes such as glucan synthesis, cell death and photosynthesis. We also identified regulated transcription factor families, including WRKY, CAMTA, GRAS and ZIM. These results delineate the metabolic events associated with regulation of the PA biosynthetic pathway in the presence of *lpa*-causing mutations. We also quantified transcript levels of genes involved in the raffinose family oligosaccharides pathway in *3mlpa* mutant. Overall, these results contribute to an understanding of regulation of metabolism in *3mlpa* mutant during seed development.

## Methods

### Genetic material and background

The experimental lines of soybean (*Glycine max* (L.) Merr.) used in this study were: (a) a triple homozygous mutant line, designated as ‘3*mlpa*’ (*mips1/mrp-l/mrp-n*) with *lpa*, and (b) a sibling line with homozygous non-mutant alleles, designated as ‘3MWT’ (MIPS1/MRP-L/MRP-N) with normal PA (Table 1). These lines were developed from a cross of V99-5089 with CX-1834 [16]. V99-5089 soybean experimental line carries a point mutation in MIPS1 gene (chromosome 11) that results in *lpa*, low stachyose, and high sucrose phenotype [15]. Similarly, CX-1834 soybean line carries point mutations in two MRP genes, namely, MRP-L and MRP-N, located on chromosomes 19 and 3 respectively. Both *mrp* mutations are required to obtain the *lpa* phenotype, and have no effect on seed stachyose and sucrose contents [12, 16, 63]. The MIPS1 gene does not interact with MRP-L and MRP-N genes. Although the *3mlpa* line, carrying mutations in MIPS1 and two MRP genes, shows reduced PA content, the stachyose and sucrose phenotype associated with the *mips1* mutation was rescued (Table 1) [41]. The low phytic acid soybean exhibits reduced seed emergence, however, the molecular basis of this relationship is still unknown.

### Plant growth, and sampling

For each of the two experimental lines, 48 plants were grown in 12 pots (four plants per pot) containing Metro-Mix® 360 (Sun Gro) soilless media, over-layered with topsoil GardenPro ULTRA^LITE^. All plants were grown in the same growth chamber unit, with controlled conditions, as follows: 14/10 h (day/night) photoperiod, 24/16 °C (day/night), light intensities in the range of 300 and 400 μE and 50–60 % relative humidity. About 41–47 plants from each experimental line were used for sampling developing seeds. Seed length was the criterion for sampling different developmental stages. Pods were randomly selected, opened and, seed length was measured. Five developmental stages were defined by seed length as: (Stage 1) between 2–4 mm; (Stage 2) between 4–6 mm; (Stage 3) between 6–8 mm; (Stage 4) between 8–10 mm; and, (Stage 5) between 10–12 mm (Fig. 1). Three biological replicates for each stage were taken, where each replicate sample was represented by a minimum of 10–15 seeds (stages 1–2), and at least 3 seeds (stages 3–5), collected from different pods on separate plants. Sampled seeds were immediately frozen in liquid nitrogen and stored at **−**70 °C.

### RNA extraction, library preparation, and mRNA sequencing

Frozen seeds were ground to a fine powder and total RNAs were extracted using RNeasy Plant Mini Kit, with on column DNase digestion (QIAGEN). RNA concentrations were determined by UV spectrophotometry (260 nm, NanoDrop 1000, Thermo Fischer Scientific). RNA concentrations were normalized to 200 ng/μl and the RNA integrity number (RIN) was measured using a Bioanalyzer (Agilent Technologies). Total RNA samples with RIN values ranging between 9.0–10.0, an indication of high quality, were kept. High quality total RNA samples (50 μl each) were used for library preparation and mRNA sequencing at the Génome Québec Innovation Centre, Canada. A total of 30 cDNA libraries were generated using the TruSeq RNA sample preparation kit (Illumina) and sequenced on five lanes of a HiSeq2000 sequencing system (Illumina), to obtain single-end 100-bp long RNA-seq reads. Six libraries representing three biological replicates of single sampling stage from both *3mlpa* and 3MWT were multiplexed together in single lane.

### Transcriptomics data processing and analysis

Sequencing data quality control was performed prior to data analysis. Sequencing reads were then mapped/aligned to the well-annotated ‘Williams 82’ soybean reference genome (assembly Glyma.Wm82.a1.0, annotation v1.1) using the splice-aware mapping tool, TopHat, v2.0.8 [42, 64, 65]. Sequence mapping data was used to estimate expression values for annotated genes using HTSeq-count ‘Union’ mode [66]. Differential gene expression analyses were performed using the statistical tool, DESeq, v1.12.1 [67]. Fold-change (FC) was calculated by dividing mean normalized gene expression value in *3mlpa* over that in 3MWT. Most significant genes were identified at 1 % false discovery rate (FDR) calculated using P-value adjusted for multiple testing using Benjamini-Hochberg method [52]. Functional enrichment analysis was performed to identify ontology terms and pathways represented by these significant genes using online AgriGO tool [68]. The enriched results were then filtered using 1 % FDR to obtain highly significant enriched terms. R-script for statistical hyper-geometric test was used to identify significantly enriched transcription factor families. Most significant GO terms and transcription factor families were identified at 1 % FDR using Benjamini-Hochberg method.

### Quantitative real time PCR

RNA-Seq output was validated using quantitative PCR (qPCR). First strand cDNA was synthesized from two μg high quality total RNA (see above) using High Capacity RNA-to-cDNA kit (Applied Biosystems) following manufacturer’s instructions, from a total of 30 samples comprising three biological replicates for each time point. Two μl of stock cDNA from each of the 30 samples was diluted ten fold in water. Quantitative real time PCR was performed in 20 μl total reaction volume, constituted of four μl of diluted cDNA, 10 μl of 2× SYBR Green PCR Master Mix (Applied Biosystems), 0.4 μl of 10 mM each gene-specific primers, and 3.2 μl of UltraPure™ distilled water. PCR conditions were: 50 °C for 2 min, 95 °C for 10 min, followed by 40 cycles at 95 °C for 20 s and primer annealing temperature for 1 min, using 7500 Real Time PCR system (Applied Biosystems). Melting curve analyses were performed to test primer specificity. Target gene specific primers were designed according to the soybean reference sequence using Primer3.0 for six randomly selected most significant differentially expressed genes [69, 70]. Primer information is provided in Additional file 7. Ubiquitin 10 (UBQ10) gene was used as a reference gene to normalize target gene transcript level amongst all samples [71]. For estimating target and reference gene efficiency, equal volumes of diluted cDNA from all samples were pooled together. Standard curve of Ct values was generated using five-fold serial dilution of this pooled cDNA sample. PCR efficiency was estimated using slope of this standard curve as: *E* = 10^(−1/*slope*)^. Relative quantification of target gene transcript was estimated using Pfaffl (or, efficiency correction) method [72]. The fold change gene expression values of stage-specific samples estimated using qPCR and RNA-Seq analyses were first tested for normality and to compare variance using the Shapiro-Wilk test and F-test, respectively. The Student’s t and Mann-Whitney-Wilcoxon tests were performed to check the null hypothesis that there is no significant difference in qPCR and RNA-Seq data at the significance level of 0.01 (Additional file 8).

### Availability of supporting data

The data sets supporting the results of this article are available in the NCBI-Gene Expression Omnibus (GEO, http://www.ncbi.nlm.nih.gov/projects/geo/) accession no. GSE75575.

## Additional files

Additional file 1:
**Sequencing data from developing seed tissue of soybean.** (XLSX 57 kb) Additional file 2: Figure S1. Relative gene expression of MIPS1, MRP-L, MRP-N. Fold change between *3mlpa* and 3MWT at respective seed developmental stages for genes encoding (a) MIPS1, (b) MRP-L and (c) MRP-N. Green and orange bars indicate mean fold change values from RNA-Seq and qPCR experiments, respectively. There was no significant difference in the gene expression profiles estimated using qPCR and RNA-Seq analyses at significance level of 0.01 (See Additional file 7 for more information) (PDF 27 kb) Additional file 3:
**Defense related genes differentially expressed in early stages of seed development.** Column ‘S’ reports the stages of development in which the gene is differentially expressed. Genes down-regulated in *3mlpa* mutant are indicated by negative values of *log*_*2*_ ratio. Adjusted P-values are only reported for the stages in which the gene is differentially expressed. (XLSX 60 kb) Additional file 4:
**Photosynthesis related genes differentially expressed in late stages of seed development.** (XLSX 49 kb) Additional file 5:
**Cellular glucan metabolism related genes differentially expressed in early stages of seed development.** The genes up-regulated in 3mlpa mutant in both stages are indicated by asterisk (*) symbol. The genes differentially expressed only in stage 1 and stage 2 are indicated by section sign (§) and double dagger (‡) symbols, respectively. The non-significant adjusted P-values are indicated by dash (-). The genes down-regulated in 3mlpa mutant are indicated by negative values of log2 ratio. Zero read count (no gene expression) in 3MWT, resulting in infinite value for FC and log2 ratio indicated by infinite sign (∞). (XLSX 59 kb) Additional file 6:
**Enriched multidrug transporter genes up-regulated in** ***3mlpa (XLSX 51 kb)***
Additional file 7:
**Primers used for quantitative real-time PCR.** Gene ID for MIPS1 was not available for the first reference soybean assembly; therefore it is indicated as ‘NA’. Published primer sequences were obtained for MIPS1 [18], RS2 [60] and housekeeping gene, UBQ10 [71] (XLSX 45 kb) Additional file 8:
**Statistical test results for qPCR and RNA-Seq data comparison.** The stage-specific data for each gene was tested for normality and sample variances using Shapiro-Wilk’s test and F-test, respectively. This was followed by Student’s *t*-test and Mann-Whitney *U* test to check the null hypothesis that there is no significant difference in the gene expression data estimated using qPCR and RNA-Seq analyses, at the significance level of 0.01. Samples that showed significant difference in the expression between qPCR and RNA-Seq data are indicated by asterisk (*) symbol. The sample for which qPCR and RNA-Seq data showed unequal variance is indicated by hash (#) symbol (XLSX 51 kb)

## Abbreviations

PA: Phytic acid
*Lpa*: Low phytic acid
MIPS1: *myo*-inositol phosphate synthase 1
MRP: Multidrug resistant protein
RNA-Seq: mRNA sequencing
PCA: Principal component analysis
DEG: Differentially expressed gene
GO: Gene ontology
TF: Transcription factor
CESA: Cellulose synthase
XET: Xyloglucan endotransglucosylase/hydrolase
SugT1: Sugar transporter
GRAS: Gibberellin-insensitive, repressor of ga1–3, scarecrow
ZF-HD: Zinc finger-homeodomain
ZIM: Zinc-finger protein expressed in inflorescence meristem
CAMTA: Calmodulin-binding transcription activator
GRF: Growth-regulating factor1
MBF1: Multiprotein bridging factor 1
TCP: Teosinte branched 1, cycloidea, PCF
LRR: Leucine rich repeat
NB-ARC: Nucleotide-binding adaptor APAF-1, R proteins, and CED-4
ADR1-L1: Activated disease resistance 1-like 1
ROS: Reactive oxygen species
SA: Salicylic acid
PSI: Photosystem I
PSII: Photosystem II
LHC: Light harvesting complex
CHLM: Magnesium protoporphyrin IX methyl transferase
MFS: Major facilitator superfamily
MATE: Multidrug and toxic compound extrusion
ABC: ATP binding cassette
PGP: P-glycoprotein
RFO: Raffinose family oligosaccharide
GS: Galactinol synthase
RS: Raffinose synthase
SS: Stachyose synthase
