# Supplementary figures and images for: Genome-wide transcriptome analyses of developing seeds from low and normal phytic acid soybean lines
Source: BMC Genomics. 2015 Dec 18;16:1074. doi: 10.1186/s12864-015-2283-9 (PMC4683714; doi:10.1186/s12864-015-2283-9)

(a) MIPS1

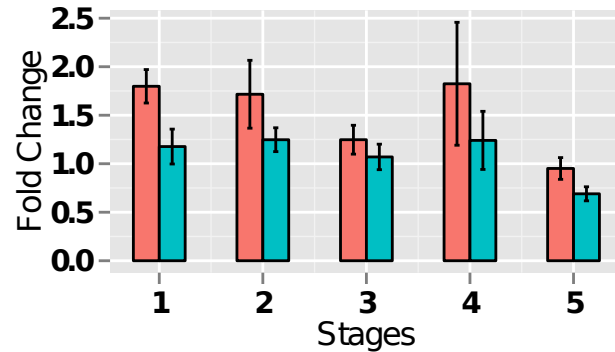

(b) MRP-L

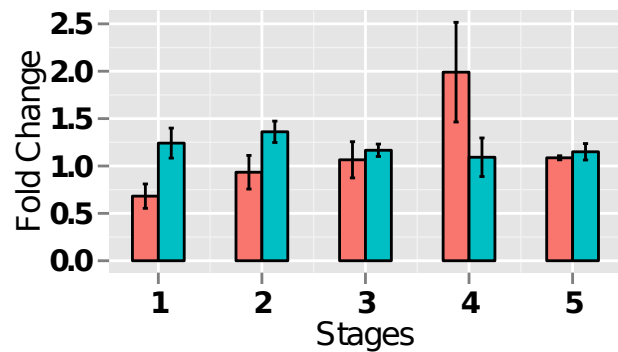

(c) MRP-N

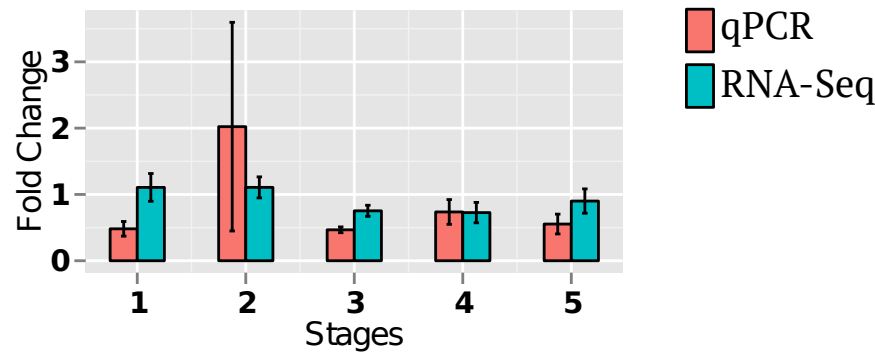

Supplement: Additional file 2: Figure S1. — Relative gene expression of MIPS1, MRP-L, MRP-N. Fold change between 3mlpa and 3MWT at respective seed developmental stages for genes encoding (a) MIPS1, (b) MRP-L and (c) MRP-N. Green and orange bars indicate mean fold change values from RNA-Seq and qPCR experiments, respectively. There was no significant difference in the gene expression profiles estimated using qPCR and RNA-Seq analyses at significance level of 0.01 (See Additional file 7 for more information) (PDF 27 kb) [file 12864_2015_2283_MOESM2_ESM.pdf]
